# Differences in medication adherence are associated with beliefs about medicines in asthma and COPD

**DOI:** 10.1186/s13601-017-0175-6

**Published:** 2017-11-10

**Authors:** Susanne Brandstetter, Tamara Finger, Wiebke Fischer, Magdalena Brandl, Merle Böhmer, Michael Pfeifer, Christian Apfelbacher

**Affiliations:** 1 Medical Sociology, Institute of Epidemiology and Preventive Medicine, University of Regensburg, Dr.-Gessler-Str. 17, 93051 Regensburg, Germany; 2 Department of Public Health Microbiology and Infectious Disease Epidemiology, Bavarian Health and Food Safety Authority, Oberschleissheim, Germany; 3 Department of Pneumology, Klinik Donaustauf, Donaustauf, Germany

**Keywords:** Medication adherence, Compliance, Beliefs about medicines, Asthma, COPD, Chronic obstructive lung diseases

## Abstract

Adherence to medication is crucial for achieving treatment control in chronic obstructive lung diseases. This study refers to the “necessity-concerns framework” and examines the associations between beliefs about medicines and self-reported medication adherence in people with chronic obstructive lung disease. 402 patients (196 with asthma, 206 with COPD) participated in the study and completed a questionnaire comprising the “Beliefs about Medicines-Questionnaire” (BMQ) and the “Medication Adherence Report Scale” (MARS). Multivariable logistic regression analyses with the BMQ-subscales as explanatory and the dichotomized MARS-score as dependent variable were computed for the asthma and the COPD sample, respectively, and adjusted for potentially confounding variables. 19% of asthma patients and 34% of COPD patients were completely adherent to their prescribed medication. While specific beliefs about the necessity of medicines were positively associated with medication adherence both in patients with asthma and with COPD, general beliefs about harm and overuse of medicines by doctors were negatively associated with medication adherence only among patients with asthma. The findings of this study suggest that patients’ specific beliefs about the necessity of medicines represent an important modifiable target for improving patient–doctor consultations when prescribing medicines.

## Introduction

Medication adherence is considered crucial in the treatment of chronic conditions. Non-adherence or insufficient adherence affect the effectiveness of therapies negatively and increase both morbidity and health care costs [1–3]. Estimates suggest that only about half of people with chronic conditions in industrialized countries are adherent to their prescribed medication [4].

Research on predictors of medication adherence has largely yielded inconsistent findings, in particular with regard to the associations between medication adherence and socio-demographic or disease related characteristics [5, 6]. The “necessity-concerns framework” adopts another perspective: This theoretical model seeks to explain medication adherence by emphasizing the importance of beliefs people hold regarding their medication [7, 8]. Strong beliefs about the necessity of medicines are assumed to affect adherence in a positive way, while concerns regarding potential side effects or harm might hinder medication adherence. Over recent years many studies have employed the necessity-concerns framework including numerous patient samples with a variety of chronic conditions [9].

Chronic obstructive lung diseases are of particular relevance for research on medication adherence as medical treatment holds special requirements for patients: therapeutic regimes often comprise not only various medications, but also different ways of application (including pills and inhalers). Inhalers are hard to manage for many patients and much research has investigated patients’ difficulties regarding the correct handling of inhaler devices [10]. Medication non-adherence is even more common than in other chronic diseases [6] and has been shown to be associated with a deterioration of clinical outcomes in patients with COPD (chronic obstructive pulmonary disease) [11] and with asthma [12]. Several studies in patients with asthma have used the necessity-concerns framework to investigate adherence [e.g. 13–15]. However, only one study was conducted in people with COPD [16].

This study aimed at contributing to the body of research on beliefs about medicines and medication adherence and reports findings obtained from a sample of people with chronic obstructive lung diseases.

## Methods

### Design

This study used baseline data from a longitudinal patient cohort study [17]. Ethical approval was obtained from the Ethics committee of the University of Regensburg (File-Numbers 12-101-0162, 13-101-0091).

### Sample and recruitment

Settings for patient recruitment covered both ambulatory and stationary care in Regensburg (Eastern Bavaria, Germany) and adjacent regions: general practitioners, physicians specialized in the treatment of patients with thoracic diseases and hospitals. The first patient was included in June 2013, the last patient in December 2014.

Patients were eligible for participation in the study if asthma or COPD had been diagnosed by a physician at least 3 months ago, if they were adults, if they had sufficient knowledge of German and if they had no mental disorder (except affective disorders or anxiety). Patients were approached by study staff, informed about the aims and procedures of the study and asked to provide written informed consent.

### Data assessment and measures

Data assessment included a comprehensive self-report questionnaire and information on the currently prescribed medicines extracted from patients’ (electronic) health records. We determined the overall number of different medicines as well as the number of inhalers, pills and other medications.

Standardized, validated self-report instruments were used to assess the main constructs of the study (beliefs about medicines, medication adherence) and potential influencing factors (disease severity/control, anxiety, depression, self-efficacy, health-related quality of life). The instruments that were selected are described in the following:

The “Beliefs about Medicines-Questionnaire” (BMQ) [18] covers people’s general beliefs regarding medicines and specific beliefs regarding their prescribed medicines in 23 items on five subscales: general overuse (4 items), general harm (4 items), general utility (4 items), specific necessity (5 items) and specific concerns (6 items). The response format is a five-point Likert scale. Mean values are calculated for the five subscales ranging from 1 to 5 with higher values  indicating stronger beliefs.

Medication adherence was assessed through the “Medication Adherence Report-Scale” (MARS) [19]. It comprises unintentional and intentional aspects of adherence behaviour on a single scale with five items. Response options range from “never” to “always”. A sum score is calculated with higher values indicating a higher extent of adherence.

Disease severity and disease control were measured by means of the Asthma Control Questionnaire (ACQ: 6-items version) [20] in patients with asthma and by means of the 8-item COPD assessment test (CAT) [21] in patients with COPD. Higher scores relate to more severe symptoms or worse disease control, respectively.

The Hospital Anxiety and Depression Scale (HADS) [22] assesses symptoms of anxiety and depression in two subscales with each 7 items. Higher sum scores indicate an increasing frequency of symptoms of depression and anxiety.

Generalized self-efficacy refers to a person’s beliefs regarding his/her successful mastery of difficulties or demands in everyday living. It was measured by the Generalized Self-Efficacy Scale (GSE) [23], which comprises 10 items on one scale. A higher sum score refers to higher levels of self-efficacy.

The 12-item Short Form Health Survey (SF-12) [24] was administered to participants in order to measure health-related quality of life (HRQoL). The SF-12 allows for deriving two scores (mental and physical component score: MCS, PCS) which are transformed to a scale from 0 to 100 (with mean = 50, SD = 15) using the published population norms [25].

Additional items in the patient questionnaire addressed participants’ socio-demographic background (sex, age, migration background, marital status, education) and variables related to disease and therapy (duration of disease, experience of severe side effects, visits to physician, distance to physician).

### Statistics

Descriptive statistics were used to display patient characteristics. Group differences between the asthma and the COPD sample were analyzed using χ^2^-tests for categorical variables and *t* tests for continuous variables.

The distribution of the outcome variable (MARS score) was highly skewed towards scores indicating higher adherence levels. Thus, it was dichotomized into “completely adherent” (25 points) and “not completely adherent” (≤ 24 points).

In order to examine the associations between beliefs about medicines and medication adherence, multivariable logistic regression models were computed for all five BMQ-subscales. Results are given as odds ratios (ORs) and 95% confidence intervals (95% CIs). All potentially confounding variables that were associated with medication adherence in univariable analyses (criterion: *p* ≤ .2) were included in the multivariable regression models. Cases with missing values either in the predictor or in the outcome or in the potentially confounding variables were excluded from the multivariable regression models. All univariable and multivariable analyses were computed for the asthma sample and the COPD sample, respectively.

The analyses were computed with IBM SPSS Statistics 24.

## Results

### Patient characteristics

402 patients took part in the study: 196 had a diagnosis of asthma, 206 a diagnosis of COPD. 6 of the patients assigned to the group of COPD patients had also a concurrent diagnosis of asthma (ACOS: asthma-COPD overlap syndrome). However, both their symptoms and their prescribed medication suggested that COPD was the predominant disease. Sociodemographic, medical and psychosocial characteristics of the sample stratified for asthma/COPD are displayed in Table 1. There were significant differences between the two samples in the majority of the analyzed variables. As compared to the sample of patients with asthma, patients with COPD were more often male (*p* < .01), older (*p* < .01) and they had lower education levels (*p* < .01). Overall, they were more seriously ill as reflected by the number of prescribed medicines (*p* < .01), the number of visits to a physician (*p* < .01) and their physical and mental health-related quality of life (*p* < .01). Participants with asthma and with COPD did not differ in their general beliefs about medicines, but patients with COPD held stronger beliefs about the specific necessity of medicines (*p* < .01) and had more concerns (*p* < .01).

**Table 1:** Patient characteristics: overall and stratified by disease

	All (N = 402)	Patients with asthma (N = 196)	Patients with COPD (N = 206)	*p*
Male (%)	50.5	39.8	60.7	<.01
Age (years) (M; SD)	56.7 (15.9)	47.7 (16.7)	65.3 (8.85)	<.01
Migrational background (%)	6.0	7.1	5.0	.36
Living alone (%)	19.3	16.3	22.3	.13
Schooling				<.01
< 10 years (%)	57.5	40.3	74.4	
10 years (%)	26.7	33.0	20.5	
≥ 10 years (%)	15.8	26.7	5.1	
Age at diagnosis (years) (M; SD)	46.7 (17.8)	36.4 (18.3)	56.6 (10.1)	<.01
Duration of disease (years) (M; SD)	9.9 (9.1)	11.5 (9.9)	8.4 (7.9)	<.01
Inpatient treatment (%)	37.8	4.6	69.4	<.01
Time needed to visit provider of asthma/COPD treatment (min) (M; SD)	27.2 (17.6)	24.2 (16.7)	30 (18.0)	<.01
Number of visits to the provider of asthma/COPD treatment during the last year (M; SD)	4.7 (5.2)	3.0 (2.4)	6.3 (6.5)	<.01
Disease severity/control				n.a.
CAT score	–	–	23.2 (7.2)	
ACQ score	–	1.5 (1.2)	–	
Experience of severe side effects (% yes)	16.4	8.3	24.2	<.01
Number of prescribed medicines (M; SD)	4.8 (3.9)	2.7 (2.3)	6.9 (4.0)	<.01
Number of prescribed inhalers (M; SD)	1.9 (0.9)	1.7 (0.9)	2.1 (1.0)	<.01
Number of prescribed pills (M; SD)	2.7 (3.5)	0.8 (1.9)	4.5 (3.6)	<.01
Number of other prescribed medicines (M; SD)	0.2 (0.6)	0.1 (0.4)	0.4 (0.7)	<.01
SF-12 PCS (M; SD)	39.0 (12.6)	46.5 (10.8)	31.8 (9.7)	<.01
SF-12 MCS (M; SD)	40.9 (13.7)	44.0 (12.4)	38.0 (14.3)	<.01
GSE (M; SD)	24.4 (6.0)	30.7 (5.0)	28.2 (6.6)	<.01
HADS-depression (M; SD)	6.7 (4.7)	5.2 (4.1)	8.3 (4.6)	<.01
HADS-anxiety(M; SD)	7.8 (4.3)	7.4 (4.2)	8.1 (4.3)	.10
BMQ- overuse (M; SD)	3.1 (0.7)	3.1 (0.7)	3.2 (0.7)	.68
BMQ- harm (M; SD)	2.4 (0.7)	2.4 (0.6)	2.5 (0.7)	.15
BMQ- utility (M; SD)	3.9 (0.7)	3.9 (0.6)	3.9 (0.7)	.61
BMQ- concerns (M; SD)	2.4 (0.8)	2.2 (0.7)	2.6 (0.8)	<.01
BMQ- necessity (M; SD)	3.8 (0.9)	3.5 (1.0)	4.1 (0.7)	<.01

Group differences were analysed using χ^2^ or *t* tests

*CAT* COPD assessment test, *ACQ* Asthma Control Questionnaire, *SF*-*12 PCS* Physical Component Scale, *SF*-*12 MCS* Mental Component Scale, *GSE* Generalized Self-Efficacy Scale, *HADS* Hospital Anxiety and Depression Scale, *BMQ* Beliefs about Medicines-Questionnaire

### Medication adherence

**Fig. 1:**
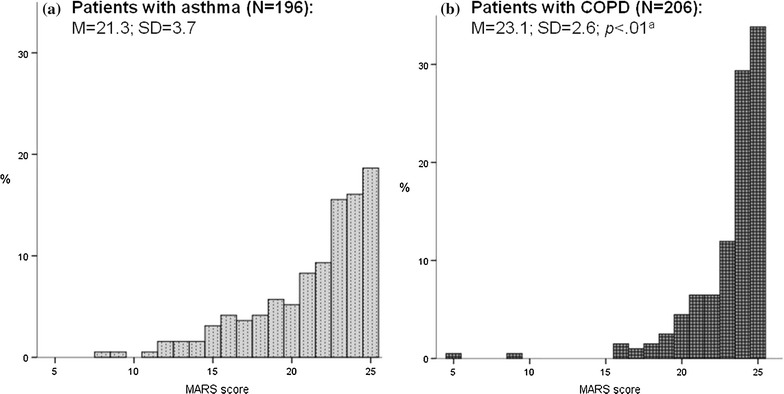
Distribution of the MARS score in the asthma sample (**a**) and in the COPD sample (**b**). MARS: Medication Adherence Report Scale. Sum score derived from five items, with higher values indicating higher medication adherence (range 5–25). ^a^Group difference was analyzed using a *t* test

The extent of patients’ medication adherence behaviour is depicted in Fig. 1. According to the MARS, the mean medication adherence was significantly lower in patients with asthma as compared to patients with COPD (*p* < .01). The dichotomization of the MARS score in patients who are completely adherent versus not completely adherent results in 19% of asthma patients and 34% of COPD patients being completely adherent to their medication.

### Beliefs about medicines and medication adherence

In the sample of patients with asthma, age, education, the numbers of prescribed inhalers and pills, the distance to the provider of asthma treatment, disease control (ACQ score) and symptoms of anxiety and depression (HADS) were associated with medication adherence in univariable analyses (criterion *p* ≤ .2) and, thus, were included in the multivariable logistic regression models (see Table 2). Patients’ general beliefs about overuse and harm of medicines as well as patients’ specific beliefs about the necessity of medicines were significantly associated with medication adherence. The chance of being adherent was reduced to about 40% in patients who held stronger beliefs about medication overuse by doctors (OR 0.42, 95% CI 0.22–0.80) and about harm of medicines (OR 0.43, 95% CI 0.21–0.88), while patients with stronger beliefs about the necessity of their medicines were three times as likely to be completely adherent (OR 2.97, 95% CI 1.54–5.73).

**Table 2:** Logistic regression analyses of medication adherence on beliefs about medicines in patients with asthma

	Not adjusted			With adjustment^a^		
	OR	95% CI	*p*	OR	95% CI	*p*
BMQ-subscales						
*General beliefs*						
Overuse	0.56	0.34–0.93	.02	0.42	0.22–0.80	<.01
Harm	0.55	0.30–1.01	.05	0.43	0.21–0.88	.02
Utility	0.40	0.80–2.78	.21	1.39	0.68–2.82	.36
*Specific beliefs*						
Necessity	2.04	1.29–3.22	<.01	2.97	1.54–5.73	<.01
Concerns	0.83	0.50–1.38	.47	0.68	0.37–1.28	.24

*OR* odds ratio, *95% CI* 95% confidence interval, *BMQ* Beliefs about Medicines-Questionnaire, *MARS* Medication Adherence Report-Scale

^a^Models adjusted for age, education, number of prescribed inhalers, number of prescribed pills, distance to provider of asthma treatment, ACQ score (Asthma Control Questionnaire) score, HADS (Hospital Anxiety and Depression Scale) depression score, HADS anxiety score adjusted models: N: 173-177; Nagelkerke’s R^2^: .31–.39

Table 3 summarizes the results of the logistic regression analyses of medication adherence on beliefs about medicines in patients with COPD. Being in inpatient or outpatient treatment, disease severity (CAT score), symptoms of anxiety and depression (HADS), generalized self-efficacy and both the physical and the mental component scale of the SF-12 were associated with medication adherence in univariable analyses. Patients’ specific beliefs about the necessity of medicines were significantly associated with medication adherence (OR 2.46, CI 1.36–4.42), with stronger beliefs increasing the chance for adherence. The other BMQ-subscales were not significantly associated with adherence in the adjusted models.

**Table 3:** Logistic regression analyses of medication adherence on beliefs about medicines in patients with COPD

	Not adjusted			With adjustment^a^		
	OR	95% CI	*p*	OR	95% CI	*p*
BMQ-subscales						
*General beliefs*						
Overuse	0.70	0.47–1.06	.09	0.61	0.37–1.00	.05
Harm	0.81	0.52–1.25	.34	0.87	0.53–1.42	.58
Utility	1.29	0.84–1.98	.25	1.40	0.85–2.30	0.18
*Specific beliefs*						
Necessity	1.56	1.00–2.44	.05	2.46	1.36–4.42	<.01
Concerns	0.64	0.43–0.97	.03	0.86	0.53–1.41	.55

*OR* odds ratio, *95% CI* 95% confidence interval, *BMQ* Beliefs about Medicines-Questionnaire, *MARS* Medication Adherence Report-Scale

^a^Models adjusted for inpatient/outpatient treatment, CAT score (COPD assessment test), HADS (Hospital Anxiety and Depression Scale) depression score, HADS anxiety score, GSE (Generalized Self-Efficacy Scale) score, SF-12 PCS (Physical Component Scale), SF-12 MCS (Mental Component Scale) adjusted models: N: 164-171; Nagelkerke’s R^2^: .09–.16

## Discussion

While beliefs about the specific necessity of patients’ prescribed medicines were associated with medication adherence behaviour in our study, concerns about medicines were not. This finding applies both to patients with asthma and COPD and does not fully support the assumptions of the necessity-concerns framework. It could be assumed that even if medications are considered harmful or prone to evoke side effects the majority of patients with asthma/COPD do not translate these beliefs into actual behaviour.

The lack of an association between negative beliefs (i.e. concerns) and adherence among patients with asthma has already been shown by other researchers: A systematic review on determinants of adherence in patients with asthma identified 17 studies which assessed patients’ concerns of which only 9 succeeded in demonstrating the expected association [5]. With respect to patients with COPD, this finding is novel. The only other study investigating the necessity-concerns framework in a sample of patients with COPD yielded a significant association between patients’ specific concerns about medicines and medication adherence [16].

With regard to patients’ general beliefs about medicines the associations with medication adherence differed between patients with asthma and with COPD. Albeit the associations between medication adherence and patients’ beliefs regarding overuse of medicines by doctors, potential harm and utility of medicines were in the expected direction in both patient groups significant associations were found only in patients with asthma: Those were more likely to behave completely adherently if they held lower beliefs in the overuse of medicines by doctors and in harm of medicines in general. Possible explanations for the differences between patients with asthma and with COPD that refer to differences in socio-demographic and disease-related characteristics between asthma and COPD can be excluded since the analyses were controlled for variables which could potentially confound the relationship between beliefs about medicines and medication adherence. Differences in the prevalence of medication adherence between the two samples are more likely to cause this finding. In our study, persons with asthma behaved less frequently completely adherent as compared to persons with COPD resulting in a more equal distribution of the two MARS categories (adherent vs. not completely adherent) and higher statistical power.

This study has some limitations: Due to its cross-sectional design no conclusions on causality can be drawn. Furthermore, medication adherence behaviour was assessed using a self-report instrument. This could have led to a biased estimation of the extent of medication adherence. In order to reduce bias induced by social desirability the oral instruction that preceded the administering of questionnaires to study participants included information on confidentiality of individual study data. It was particularly stressed that the patients’ physicians will not be aware of any of the information given in the questionnaires. The high overall prevalence of non-adherence in our study suggests that social desirability did not hinder patients to admit also non-adherent behaviours. However, concerns regarding the precision of the MARS in measuring adherence cannot be ruled out [26].

Our study succeeded in including a heterogeneous group of patients recruited from various health care settings. Suffering from additional comorbidities was no reason for the exclusion of patients from participation in the study; this is reflected in the high number of currently prescribed medications. Even if this sampling strategy contributed to the external validity of our study it comes along with a further limitation: The heterogeneity of practices in both the establishment and the documentation of diagnoses impeded the use of data on diagnostic subtypes (such as atopic and non-atopic asthma) and comorbidities. As compared to other studies that focused on the assessment of adherence to a specific therapeutic treatment (e.g. inhaler therapy), we operationalized medication adherence as adherence to all medications which have been prescribed to a person. This approach corresponds far better to the reality of health care for patients with chronic obstructive lung diseases.

To conclude, this study demonstrated high rates in medication non-adherence in patients with chronic obstructive lung disease. Beliefs in the specific necessity of prescribed medications were found to be associated with medication adherence both in patients with asthma and with COPD and suggest an important and modifiable target for improving patient–doctor consultations when prescribing medicines. However, our findings need to be corroborated by longitudinal research first.

## Abbreviations

ACQ: Asthma Control Questionnaire
BMQ: Beliefs about Medicines-Questionnaire
CAT: COPD assessment test
CI: confidence interval
COPD: chronic obstructive pulmonary disease
GSE: Generalized Self-Efficacy Scale
HADS: Hospital Anxiety and Depression Scale
M: Mean
MARS: Medication Adherence Report Scale
OR: odds ratio
SD: standard deviation
SF-12: short form health survey
